# Why does research matter?

**Published:** 2023-01-30

**Authors:** Victor H Hu

**Affiliations:** Assistant Clinical Professor: International Centre for Eye Health, London School of Hygiene & Tropical Medicine and Consultant Ophthalmologist: Mid Cheshire NHS Hospitals, UK.

## Abstract

A working knowledge of research – both how it is done, and how it can be used – is important for everyone involved in direct patient care and the planning & delivery of eye programmes.

**Figure:**
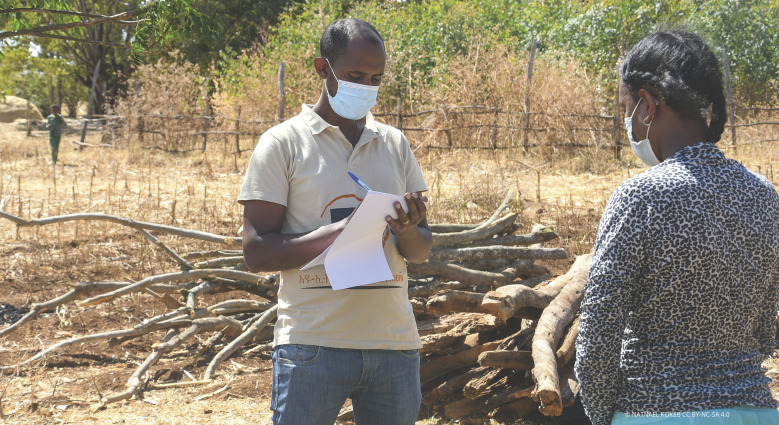
A research coordinator collecting data from a health extension worker. ethiopia

The mention of ‘research’ can be off-putting and may seem irrelevant in the busy environment of a clinic or hospital. However, research is central to all aspects of eye care delivery – both inside and outside the clinic.

Whether we are health workers, public health practitioners, managers, policy makers, or editors – all of us ‘stand on the shoulders of giants’: we rely on the research done by others before us. This can be as simple – and profound – as hand washing between patients; a habit that only became common practice in the 1870s, following the work of the Hungarian physician Ignaz Semmelweis and Scottish surgeon Joseph Lister. Or it can be as complex as making a diagnosis of glaucoma and knowing what treatment to give. All current eye care practice is based on research. Clinical, operational (eye care delivery) and public health practice will continue to be profoundly shaped by new research developments.

## What is research?

In its simplest form, research is about investigating the world around us to increase our knowledge, so we can work out how to do things better.

In health care, we use a scientific approach to carry out research; there is a set way of doing things that ensures research is done in a logical way, and that results are published widely, so that other people can scrutinise what has been done. This gives us confidence that the results will be useful in everyday practice.

It is important to critically evaluate research and research findings, including checking that research has been carried out in the proper way, and whether the conclusions that have been made are reasonable and justified. One of the ways in which the scientific community ensures the quality of research is through the process of peer review. Before research papers are accepted for publication in a scientific journal, they are reviewed by other researchers (peer reviewed) to check the quality of the research and the validity of the results and conclusions. Even so, the quality of published research can vary.

This is why systematic reviews and meta-analyses are so valuable: they answer important questions by identifying, evaluating, and summarising good quality evidence from a range of published research papers. Often, systematic reviews conclude that there is not enough evidence to answer a question with absolute certainty, or to produce an answer that will be applicable in different countries or health care settings. This is useful, as it gives researchers guidance about where more research is needed (see article on page 13).

But this can be a challenge for clinicians – how can we make good decisions in the absence of definitive evidence? Clinical experience is very important, but where possible this should be informed by good research – see page 6 for practical tips.

Health care practitioners and managers can also use guidance from professional bodies such as the World Health Organization. The article on page 8 explains the process by which guidelines are developed and shows why we can rely on them.

In conclusion, research is fundamental to the everyday practice of health care professionals, including eye care workers. Research allows us to find out new things and to provide better care for patients. There are many different types of research that can be carried out and these can vary enormously. It is important to ask the right question, as this will determine the type of research that is done (see page 5).

All of us can participate in research: it starts with asking questions and then going to find out the answers. The article on page 10 offers practical suggestions for carrying out small-scale research that is relevant and useful to eye care.

**Types of health research**

**Basic science** research, such as in molecular genetics or cell biology, fills the gaps in our understanding of disease mechanisms (pathogenesis).

**Clinical research** addresses how diseases in individuals can present and be diagnosed, and how a condition progresses and can be managed.

**Epidemiological research**, which is at the population level (as opposed to the individual level), answers questions about the number of people in the population who have a condition, what factors (called exposures) are causing the condition, and how it can be treated or prevented at the population level.

Going beyond epidemiology, there is also **operational and health systems research**, which focuses on how best to deliver health interventions, clinical and rehabilitation services, or behaviour change initiatives.

**Other types of research**, which are also important for public health, include health economics, social science, and statistical modelling.

Finally, **systematic literature reviews** can be very useful, as they identify and summarise the available evidence on a specific topic.

*By Clare Gilbert and GVS Murthy*

**Examples of research questions and how they have been answered**

**Can povidone iodine prevent endophthalmitis?**

In many eye departments, cataract surgery is a frequently preformed operation. One of the most serious complications is infection within the eye (endophthalmitis) which can lead to loss of vision. Several well conducted randomised controlled clinical trials have shown that instilling 0.5% aqueous povidone iodine eye drops, an antiseptic agent, before surgery reduces the risk of this devastating infection, with the first trial undertaken in 1991.^1^

**What is the best treatment for primary open-angle glaucoma?**

Chronic glaucoma can be a very difficult condition to manage, particularly when patients often only present to eye departments once they have already had significant vision loss. Eye drops which lower intraocular pressure are often prescribed; however, patients may not use the eyedrops because they are expensive, can be difficult to instil, and do not improve their vision. Surgery is an option, but patients can be reluctant to undergo surgery on their only good eye, and there can be postoperative complications. Laser treatment is another option. In a recent study in Tanzania, patients were randomly allocated to Timolol 0.5% eye drops or a form of laser called Selective Laser Trabeculoplasty (SLT).^2^ After one year, SLT was found to be superior to drops for high-pressure glaucoma.

**Why don't older adults in England have their eyes examined?**

Focus group discussions among older adults in England revealed that, despite most participants being eligible for state-funded check-ups, wearing spectacles was associated with the appearance of being frail. They were also afraid of appearing to ‘fail’ tests, and had concerns about the cost of spectacles.^3^

**How cost effective is a diabetic retinopathy screening programme?**

An economic evaluation in South Africa compared alternative interventions. Screening using non-mydriatic retinal photographs taken by a technician supervised by an ophthalmic nurse and read by a general medical officer was cost-effective and the savings made allowed the government to fund disability grants for people who went blind.^4^
